# Worth 4 Dot App for Determining Size and Depth of Suppression

**DOI:** 10.1167/tvst.9.4.9

**Published:** 2020-03-09

**Authors:** Ann L. Webber, Thomas R. Mandall, Darcy T. Molloy, Lucas J. Lister, Eileen E. Birch

**Affiliations:** 1 School of Optometry and Vision Science, Institute of Health and Biomedical Innovation, Queensland University of Technology, Brisbane, Australia; 2 Retina Foundation of the Southwest, Dallas, TX, USA; 3 UT Southwestern Medical Center, Dallas, TX, USA

**Keywords:** suppression, binocular vision, Worth 4 Dot

## Abstract

**Purpose:**

To describe and evaluate an iOS application suppression test, Worth 4 Dot App (W4DApp), which was designed and developed to assess size and depth of suppression.

**Methods:**

Characteristics of sensory fusion were evaluated in 25 participants (age 12–69 years) with normal (n = 6) and abnormal (n = 19) binocular vision. Suppression zone size and classification of fusion were determined by W4DApp and by flashlight Worth 4 Dot (W4D) responses from 33 cm to 6 m. Measures of suppression depth were compared between the W4DApp, the flashlight W4D with neutral density filter bar and the dichoptic letters contrast balance index test.

**Results:**

There was high agreement in classification of fusion between the W4DApp method and that derived from flashlight W4D responses from 33 cm to 6 m (α = 0.817). There were no significant differences in success rates or in reliability between the W4DApp or the flashlight W4D methods for determining suppression zone size. W4DApp suppression zone size strongly correlated to that determined with the flashlight W4D (rho = 0.964, *P* < 0.001). W4DApp depth of suppression measures showed significantly higher success rates (χ^2^ = 5.128, *P* = 0.043) and reliability (intraclass correlation analysis = 0.901) but no significant correlation to the depth of suppression calculated by flashlight W4D and neutral density bar (rho = 0.301, *P* = 0.399) or contrast balance index (rho = −0.018, *P* = 0.958).

**Conclusions:**

The W4DApp has potential clinical benefit in measuring suppression zone size; however, further modifications are required to improve validity of suppression depth measures.

**Translational Relevance:**

W4DApp iOS application will be a convenient tool for clinical determination of suppression characteristics.

## Introduction

The Worth 4 Dot (W4D) test was first developed to assess binocular perception by Claud Worth in 1903^1^ and remains one of the world's most frequently used clinical tools to evaluate unilateral suppression under binocular viewing conditions.^2^^–^^4^ Criticism of the test includes lack of standardization in manufacture, and in spacing and subtended visual angles of the dots.^5^ Further, the highly dissociative red/green filters may introduce artifact responses,^6^ and there can be difficulty achieving interpretable outcomes when examining young children.^4^ Several modifications to the original test design have resulted from these identified limitations.^4^ An early adaptation by Hardy^7^ led to the introduction of near point testing using a flashlight apparatus. The use of polarized targets improved interpretable response rates and significantly decreased dissociation,^2^^,^^3^ and increased success rates in young children was achieved with use of shaped targets instead of dots.^8^

The W4D test is the accepted method for assessing the presence or absence of central and peripheral fusion.^3^ The separation of dots commonly used for the 3 m “distance” test subtend 1.25° visual angle and evaluates central fusion. The separation of dots for the 33 cm “near” test subtend 6° and evaluates peripheral fusion.^3^^,^^5^^,^^9^ To further describe suppression, the W4D flashlight version is used to calculate the size of a suppression scotoma by changing the test viewing distance to vary visual angle of dot separation.^3^^,^^9^ However, this evaluation is time-consuming in clinical practice and requires visual angle calculations to determine the exact scotoma size. Additionally, varying the viewing test distances may induce a change in accommodative demand and vergence posture. Although the extent of influence of accommodation and vergence posture on suppression is not established, controlling these factors may improve the validity of W4D results. Depth of suppression can be graded clinically by introducing a calibrated neutral density filter to lower the relative brightness of targets; however, limited test-retest reliability is reported.^10^^–^^12^

Recent studies have reported a significant correlation between the severity of suppression and the severity of amblyopia, despite these conditions being previously considered separate entities.^11^^,^^13^ Furthermore, amblyopia treatment targeted at reducing suppression can significantly improve both binocular vision and monocular acuity.^11^^,^^13^^,^^14^ These findings emphasize the need to develop a more standardized tool for assessing size and depth of suppression for use in clinical practice and in clinical trials.

We developed an iOS application suppression test, the Worth 4 Dot App (W4DApp) to address the limitations of the classic Worth 4 Dot test. This software features the principle design of the W4D with additional features to easily quantify size and depth of suppression scotomas without complex calculations. The App interface is simple to operate, and the iOS format makes this tool highly accessible to all clinicians with a compatible device.

The main clinical advantage of the W4DApp is the ability to adjust dot parameters, including target separation and contrast. This allows accurate changes to visual angle separation from the same viewing distance, to assess suppression zone size while controlling any effects of accommodation and vergence. The W4DApp also determines depth of suppression measures without requiring additional filters. These size and depth measures provide a quantitative assessment of suppression severity, which can have potential benefit to clinicians in monitoring amblyopia, strabismus, or both.

Here we describe the design, success rate (testability), reliability and validity of the prototype W4DApp to quantify the size of suppression zone and depth of suppression, and suggest our future modifications.

## Methods

### Study Design

This is a proof-of-concept study that compared the determination of fusion classification by the W4DApp and Good-lite 950100 Worth 4-Dot Flashlight at different viewing distances. In those participants with abnormal sensory fusion, the success rate, reliability and validity for determination of suppression zone size, and depth of suppression were compared between the novel W4DApp and flashlight W4D.

### Participants

Characteristics of suppression were determined in 25 participants prospectively recruited for a study of clinical binocular vision tests (19 participants with abnormal binocular vision development from strabismus or amblyopia and 6 participants with normal vision development). Participants were aged from 12 years, and all had a comprehensive vision and intraocular health exam that included previous treatment history, visual acuity (electronic Early Treatment of Diabetic Study e-ETDRS), clinical stereoacuity (Randot Preschool Stereoacuity Test; Stereo Optical Co, Inc., Chicago, IL, USA), ocular alignment (unilateral and prism alternating cover test) and Worth 4 Dot response at 6 m and 33 cm. Classification criteria for normal binocular vision group were best-corrected visual acuity of 0.00 logMAR or better, and 60 sec of arc stereoacuity on Randot Preschool Stereoacuity Test, no suppression on classical 6 m Worth 4 Dot test and no known history of amblyopia or strabismus. Classification criteria for abnormal binocular vision group (BV-abnormal) were a known history of strabismus (with or without amblyopia), or amblyopia (anisometropic, strabismic or combined mechanism). Amblyopia was defined as an interocular difference in best-corrected visual acuity of 0.20 logMAR or more. No participants had coexisting general developmental, systemic, or ocular pathology or congenital abnormality.

Data were collected at the Queensland University of Technology. The study was conducted in accordance with the requirements of the Queensland University of Technology Human Research Ethics Committee. All participants provided written informed consent before the examination, and the protocol adhered to the tenets of the Declaration of Helsinki.

### Apparatus

#### W4D App

**Figure 1.**
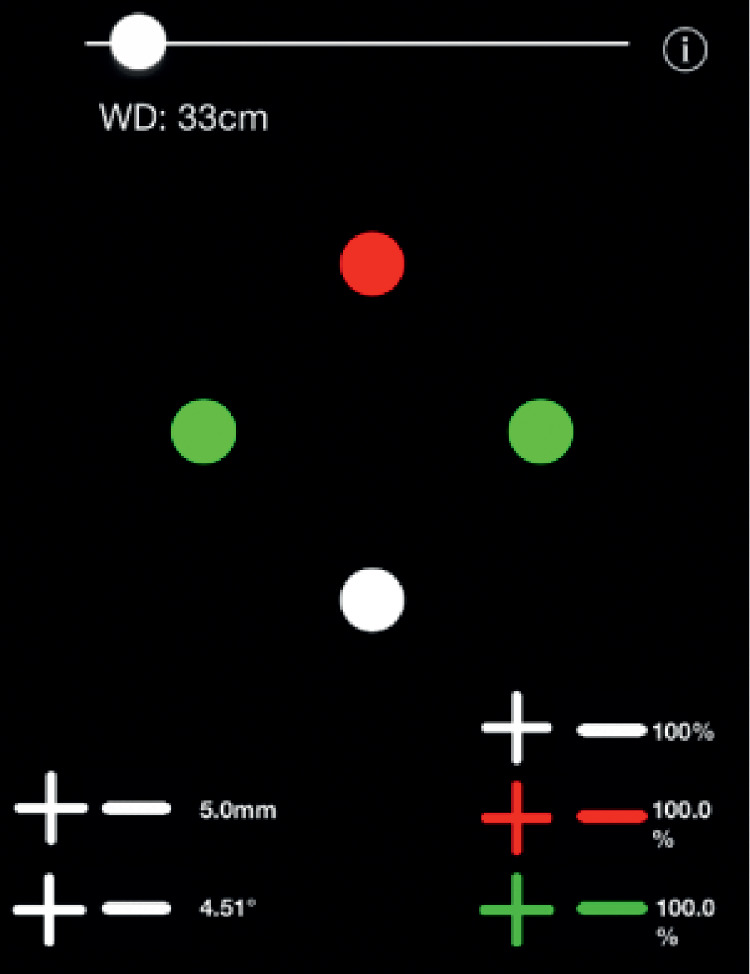
Screen shot example of W4DApp display.

The W4DApp (Fig. 1) was presented on a fifth-generation iPad (model A1822 9.7″ IPS LED-backlit 2048 by 1536 display [264 ppi]) with installed features to allow changes to dot diameter, dot separation, and color contrast. The application calculated the subtended visual angle based on the viewing distance and separation between the outside edges of two opposing dots. The presentation of target colors and shapes were set to match the standard arrangement of flashlight W4D presentation, with dimensions shown in Table 1.^2^ To generate the dots, an HSB-A color space was used, where A (Alpha) determines the opacity of the colored dot against the black background. Alpha was initially calibrated to 100%. As the alpha opaqueness value reduced, the colored dots became more transparent, resulting in reduced dot brightness. The relationship between Alpha opaqueness and measured app dot brightness was determined across a range of alpha settings. The iPad screen brightness was maintained at the maximum setting with brightness auto-adjustment turned off. As classical W4D 6-mm dots^2^ were found to overlap at small (1°) visual angles required for W4DApp bifoveal testing at 33 cm, the W4DApp dot diameter was set to 5 mm for all tests to avoid on-screen overlapping.

**Table 1.** Color and Luminance of W4D Stimulus

		Luminance (cd/m^2^)					
		W4DApp Dots			Flashlight Worth 4 Dot		
	Hue, Saturation, Brightness	Unfiltered	With Red Filter	With Green Filter	Unfiltered	With Red Filter	With Green Filter
White	0,0,0	425	63	69	620	12	55
Green	120,100,100	325	0.2	65	170	0.03	46
Red	360,100,100	85	8.9	0.3	30	5	0.06
Black surround	0,0,0	0.8			0.7		

#### Flashlight W4D

**Figure 2.**
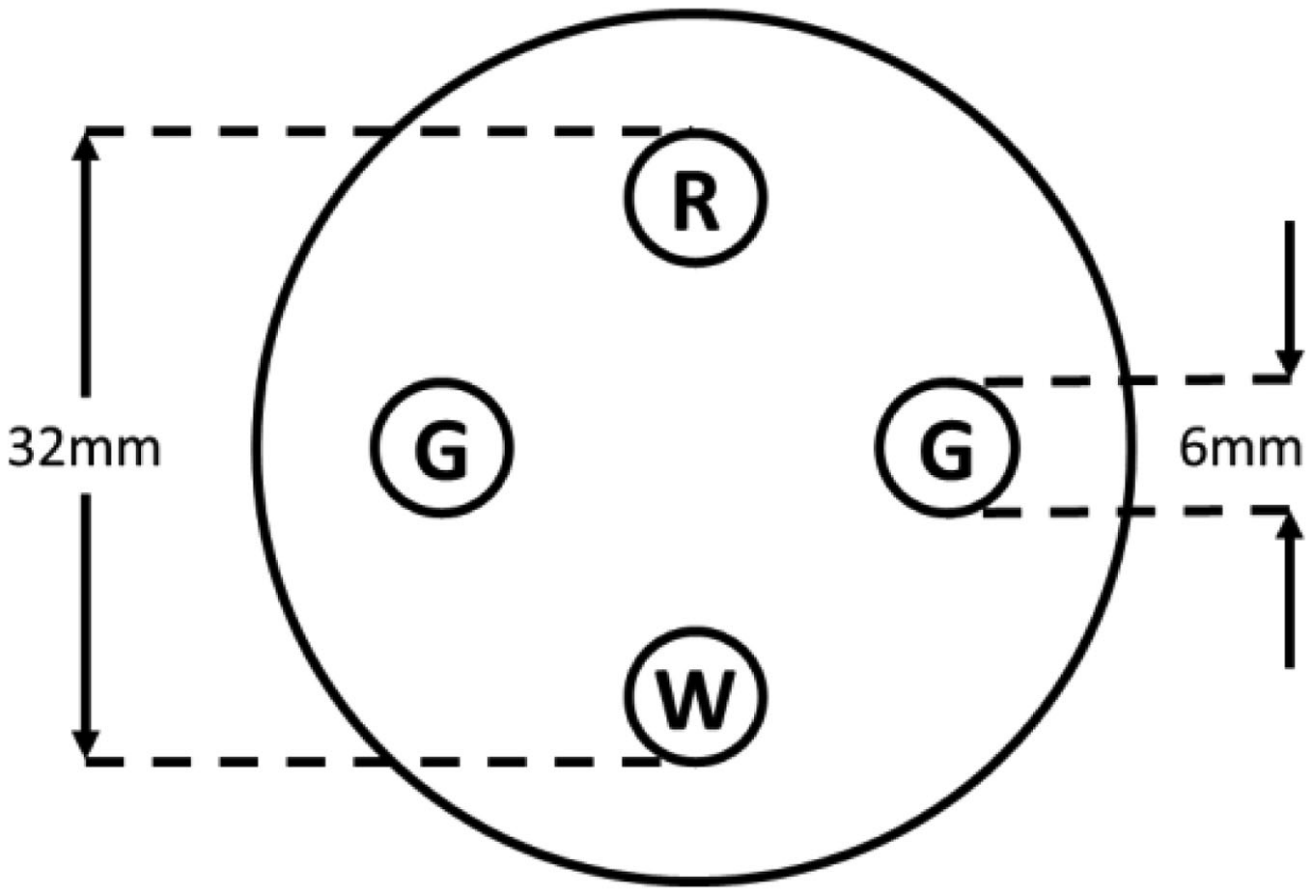
Flashlight W4D Dot parameters (Richmond Products).

The 950100 Worth 4-Dot Flashlight (Fig. 2) had a screw-on filter cap, with standard 6-mm backlit colored dots spaced equidistant around a 32-mm diameter circle (Good-Lite Company, Elgin, IL, USA).^2^ A 6-filter neutral density (NDF) bar (0.3-log-unit increments, range 0.3d–1.8d)^10^ was used in combination with the flashlight to vary light intensity of the stimulus viewed by the dominant eye.^12^ The relationship between NDF filter and light intensity was determined from 3 averaged luminance colorimeter readings of the light source through the range of filters.

#### Anaglyphic Filters

The participants wore red/green anaglyphic glasses that were supplied with the 950100 Flashlight W4D (Good-Lite Company)^2^ for both W4DApp and flashlight W4D tests, with the red filter in front of the right eye and the green filter in front of the left. The hue, saturation, and brightness of the W4DApp dots were set so that the perception of the red dot was cancelled when viewed through green lens, and the green dots were cancelled when viewed through the red lens. Stimulus parameters and luminance of the dots of the W4DApp and flashlight W4D are reported in Table 1.

#### Contrast Balance Index

A quantitative measure of depth of suppression was determined by contrast balance index (CBI), the contrast ratio between dichoptically presented letters at which each eye's letter was equally likely to be reported.^15^^–^^17^ The test displayed Sloan letters from the e-EDTRS letter set on a dichoptic 3-D monitor (5 letters per line), equivalent to 1.20 logMAR at 90-cm viewing distance. Although not an exact match, this letter size has a stroke width of 5-mm at the 90-cm viewing distance, similar to the 6-mm dot diameter of the W4D flashlight and the 5-mm dot diameter of the W4DApp. Participants wore Nvidia 3D Vision 2 Model P1431 shutter glasses that synchronized with the 3-D monitor via a Nvidia infrared emitter Model P854 (Nvidia Corporation, Santa Clara, CA, USA).

### Testing Protocol

The iPad was placed on a reading-stand positioned directly in front of the participant. The examiner input the viewing distance for App calculation of stimulus visual angle. The examiner controlled + and − buttons to vary the separation and the contrast of the dots. Dot size was held constant at 5-mm diameter. The separation of dots was incremented in 0.1° increments, and the alpha opacity was incremented in 5% steps, with the participant providing a response at each incremental change.

Testing order within size and depth measures were randomized to control for individual fatigue, and target orientations were rotated to minimize learning effects. A protocol sheet was followed to ensure all tests were performed consistently. Room illumination was controlled at 175lux throughout testing for consistency and to minimize dissociation.^3^

Suppression breakpoint was defined as the point that there was a change in participant response from suppressing (reporting 2 red or 3 green dots) to fusion or simultaneous perception (reporting 4 or 5 dots) or vice versa. For each test method, 3 breakpoints were measured using the standard W4D interpretable responses as outlined in literature,^2^^,^^4^ with participants wearing red/green anaglyph glasses.^3^ Failure to identify a suppression breakpoint was considered an uninterpretable response.

#### Size of Suppression Zone

Fusion response was classified based on suppression zone size determined by W4D responses at viewing distances 33 cm, 50 cm, 1 m, 3 m, and 6 m and by the W4DApp at 33 cm (to match standard near testing) and 90 cm (to match viewing distance of suppression depth CBI tests^13^). The size of a suppression scotoma was defined as the visual angle breakpoint where the participant reported a change in response from suppressing to seeing 4 dots. The visual angle was varied by altering viewing distance with Flashlight W4D and by changing separation of targets on the W4DApp. Fusion was classified as bifoveal for targets that fell between 0.67° and 1°, macular for targets that were within 2°–4°, peripheral for targets that were within 4°–12°, and no fusion if suppression was still reported for test distance or equivalent target separation.

#### Suppression Depth

The depth of suppression was defined as the stimulus intensity ratio between dichoptically presented targets (nondominant eye/dominant eye) where the participant reported a change in response from suppression to fusion or simultaneous perception. The W4DApp reduced intensity of dots viewed by the dominant eye by lowering the color alpha opaqueness (relative contrast). Non-dominant eye intensity remained at 100%, while dominant eye stimulus intensity was decreased. The light intensity of the Flashlight W4D dots visible to the dominant eye was reduced by introduction of neutral density filters in 0.3 log-unit increments.^10^^–^^12^ If, for example, the stimulus intensity is reduced by 50% to achieve a change in response, then the depth of suppression is 2.0 (100% divided by 50%).

CBI was derived from the contrast ratio between dichoptically presented letters at which identification of letters presented to either eye is equal (non-dominant eye contrast/dominant eye contrast).^13^ The identity and contrast of the letter presented to each eye varied, with the sum of the contrast of two overlapping letters always totaling 100%. Participants named the perceived letter in each of the 5 positions through a series of presentations, determining the contrast threshold of the left eye. Contrast threshold for the right eye was then determined by subtracting the left eye contrast threshold from one (R = 1 – L). The outcome CBI was calculated as the nondominant eye threshold divided by the fellow eye threshold. CBI of 1.0 would indicate RE and LE had equal contrast letters when it was equally likely that a RE or LE letter was reported. Higher CBI indicates that a greater difference in contrast of letters to nondominant eye was required to result in equal likelihood of reporting the dichoptic letters.

The depth of suppression ratio was determined at a 90-cm viewing distance for all 3 methods.^13^^,^^16^ Outputs from the 3 methods (W4DApp, Flashlight W4D with NDF filter, and CBI) were converted to log stimulus ratio for analysis.

### Statistical Analysis

Krippendorff's alpha was calculated to examine fusion classification agreement between test methods (KAlpha SPSS macro noting data as ordinal).^18^^,^^19^ Fisher's exact test analyses were used to identify significant differences in interpretable breakpoint response rates between size methods and between depth methods, producing exact *P*-values.^8^ Test-retest reliability was examined by performing an intraclass correlation analysis (ICC) of 3 repeated breakpoint trials for each method of suppression measurement.^20^ To compare between methods, each analysis produced an ICC value and 95% confidence intervals. Spearman's rank order correlation (rho) was used to assess the agreement between methods of determining suppression zone characteristics. Paired samples *t*-tests were used to test for difference in zone size means with 2-tail significance level. One sample *t*-tests and post-hoc regression analyses identified any bias and produced exact *P*-values. SPSS was used to perform these statistical analyses (IBM SPSS Statistics for Windows, Ver. 25.0; IBM Corp, Armonk, NY, USA).

## Results

Characteristics of sensory fusion were evaluated in 25 visually mature participants (mean age 25.2 [range 12–69] years), including n = 19 with abnormal binocular vision from amblyopia or strabismus (BV-abnormal) and n = 6 with normal binocular vision. Clinical details of BV-abnormal participants are provided in Appendix 1. Success rate, reliability, and validity were assessed in the subgroup of BV-abnormal participants who demonstrated suppression on the classical W4D test at 6 m (n = 14).

### Fusion Classification

There was absolute agreement of fusion classification between the W4D app and the Flashlight W4D for all 6 participants with normal BV (100%) and for 13 of the 19 with abnormal BV (68%) (Table 2) Krippendorff's alpha reliability estimate = 0.817 (95% CI 0.662 to 0.944). The W4D App graded 1 step smaller fusion zone in 6 of the 19 BV-abnormal participants.

**Table 2.** Classification of Fusion by iOS W4DApp and by Flashlight W4D (Number of Participants)

	Flashlight W4D at Different Viewing Distances			
	Bifoveal	Macular	Peripheral	No Fusion
W4DApp @ 33 cm				
Bifoveal	11	3	0	0
Macular	0	4	2	1
Peripheral	0	0	3	0
No fusion	0	0	0	1

### Success Rate

The success rates of the W4DApp and Flashlight W4D method were calculated as the percent of completed tests (interpretable responses) divided by the total number of attempted tests (3 attempts per participant per test), both interpretable and uninterpretable. For suppression zone size measures in the BV-abnormal group who demonstrated suppression (n = 14), there was no significant difference (χ^2^ = 0.027, *P* = 1.00) in interpretable response rates between the Flashlight W4D (34 of 42 attempts; 81.0%) and the W4DApp at 33 cm (33 of 42 attempts; 78.5%). Similarly, there was no significant difference (χ^2^ = 0.938, *P* = 0.367) in interpretable response rates between the Flashlight W4D and the W4DApp at 90 cm (37 of 42 attempts; 88.1%). For depth measures however, the W4DApp (39 of 42 attempts; 92.3%) showed significantly higher success rate (χ^2^ = 5.128, *P* = 0.043) than the Flashlight W4D (31 of 42 attempts; 73.8%).

### Reliability

All suppression zone size measures in the BV-abnormal group showed high reliability, with test-retest ICC values above 0.900 (Table 3).^20^ There was no significant difference in reliability between methods, with the Flashlight W4D ICC value within the 95% confidence intervals of the W4DApp results. However, for depth measures, the W4DApp showed significantly higher reliability than the Flashlight W4D, which showed poor reliability.

**Table 3.** Reliability: Test-retest ICC Values

Test	Single Measures ICC (2-way Random)	95% Confidence Intervals
Size Flashlight W4D	0.946	0.855 – 0.985
Size W4DApp (33 cm)	0.968	0.907 – 0.992
Size W4DApp (90 cm)	0.978	0.933 – 0.995
Depth Flashlight W4D	0.433	−0.40 – 0.829
Depth W4DApp	0.901	0.749 – 0.972

### Validity

#### Suppression Zone Size

**Figure 3.**
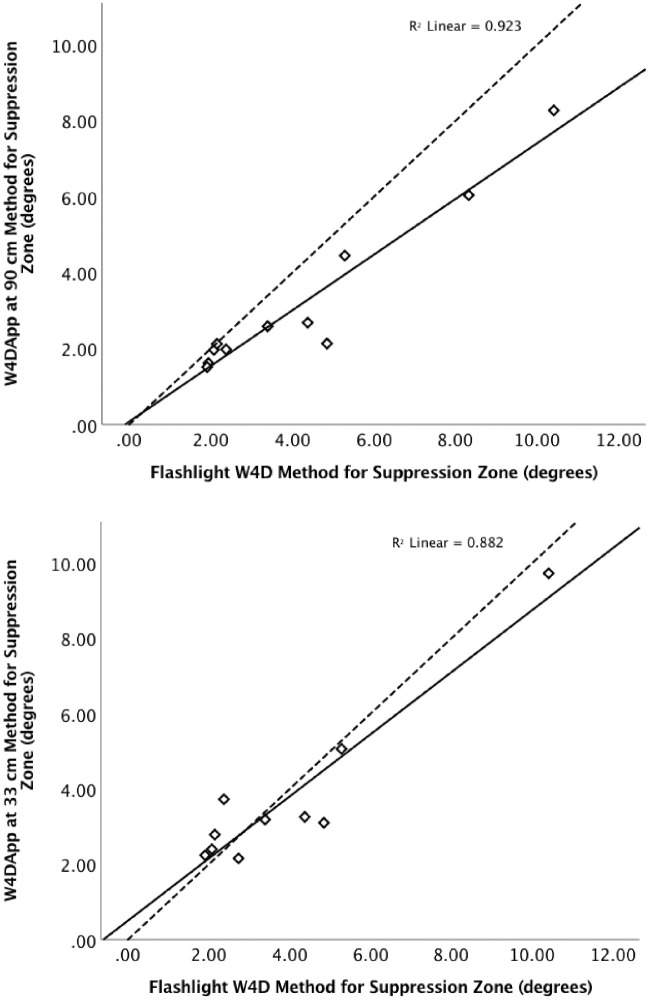
Scatterplots showing suppression zone size determined by both Flashlight W4D and W4DApp for individual participants at both 90 cm (a) and 33 cm (b). The dashed line represents absolute agreement and the solid line is the best-fit linear regression line.

Suppression zone size was successfully determined in 12 of 14 BV-abnormal participants by the W4D App at 90 cm, and successfully determined in 11 of 14 BV-abnormal participants by W4D App at 33 cm and flashlight W4D. The determination of suppression zone size by the W4DApp tested at 90 cm and the Flashlight W4D tested at range of viewing distances was highly correlated (rho = 0.964, *P* < 0.001). The mean zone size determined by W4DApp at 90 cm (3.2° ± 2.1°) was 0.9° smaller than that determined by the Flashlight W4D at different viewing distances method (4.1° ± 2.7°) (t_10_ = 3.66, *P* = 0.004). Size of zone determined by W4DApp at 33 cm also correlated with that determined by Flashlight W4D at variable distance (rho = 0.733, *P* = 0.016). However, the difference in zone size determined by the two techniques was small (3.8° ± 2.1° by W4Dapp; 4.1° ± 2.7° by Flashlight W4D method; t_9_ = 0.661; *P* = 0.525). Figure 3 presents scatterplots of individual measures of suppression zone size determined by the different methods.

#### Depth of Suppression

**Figure 4.**
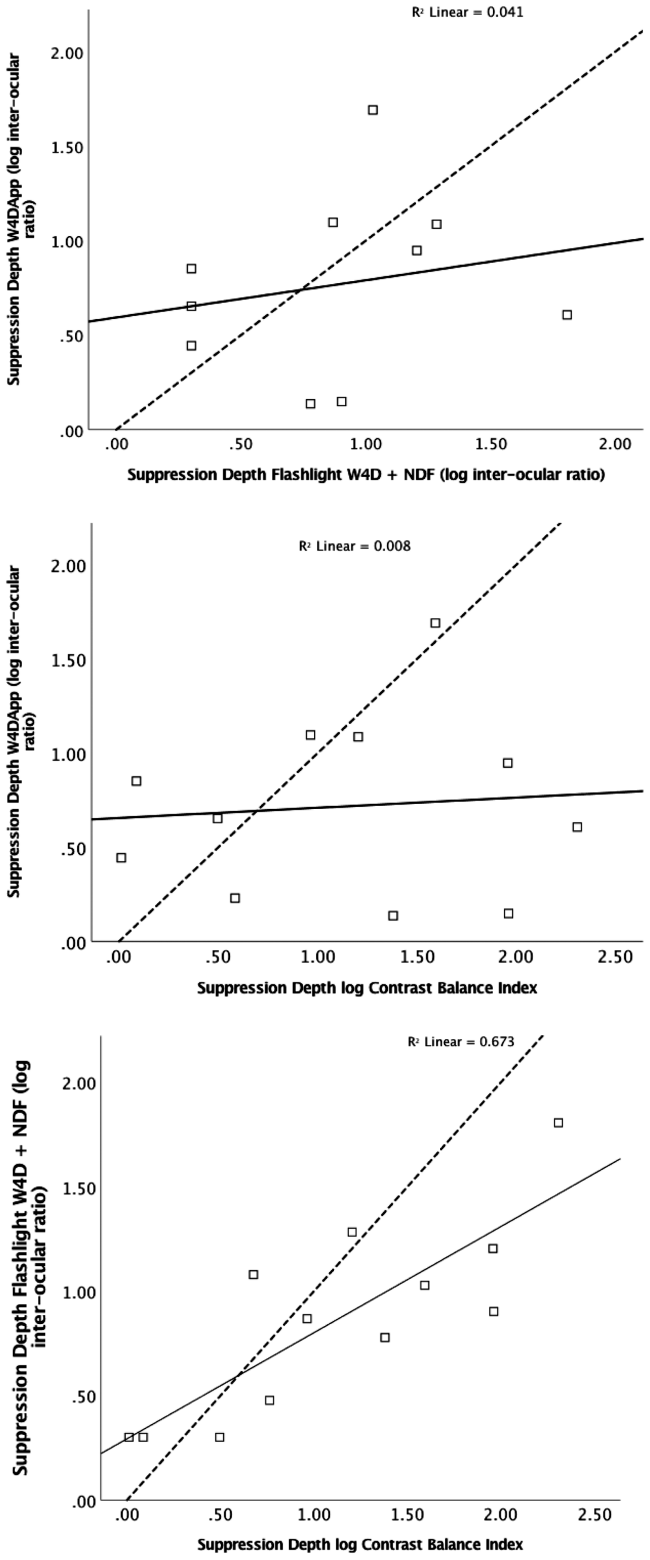
Scatterplots showing suppression depth determined by W4DApp, Flashlight W4D + NDF and Contrast Balance Index for individual participants. The dashed line represents absolute agreement between methods and the solid line is the best-fit linear regression line for relationship between methods.

Depth of suppression was successfully determined in 11 of 14 BV-abnormal participants by the W4D App, in 12 of 14 BV-abnormal participants by flashlight W4D and in all 14 BV-abnormal participants by CBI. There was no absolute agreement or significant correlation between the inter-ocular ratio for simultaneous binocular perception of dichoptically presented targets determined by W4DApp and the Flashlight W4D (rho = 0.301, *P* = 0.399) or the Contrast Balance Index (rho = −0.018, *P* = 0.958). However, significant correlation was found between the Flashlight W4D inter-ocular ratio and with the CBI (rho = 0.754, *P* = 0.005). Figure 4 presents scatterplots of individual measures of suppression depth (interocular stimulus ratio) determined by the different methods.

## Discussion

The Worth 4 Dot test is the predominant clinical method to evaluate sensory fusion, despite limitations and inconsistencies in test design.^2^^–^^5^ This report describes an iOS application version of the Worth 4 Dot test that aims to address these limitations by providing a standardized clinical tool that is highly accessible and provides accurate and meaningful clinical assessment of suppression.

In this study, we demonstrate no significant difference in success rate and reliability for the determination of suppression zone size or depth by the W4DApp at either 33 cm or 90 cm compared with outcomes determined with the Flashlight W4D. There was high agreement in classification of fusion between the W4DApp and the Flashlight W4D. Bifoveal fusion by both devices was determined in all participants with normal binocular vision development and in five participants with a history of abnormal binocular vision development from childhood anisometropia or strabismus. Suppression zone size validity analysis found that at 33 cm there was a nonsignificant average difference in zone size measurement of only 0.3° between the W4DApp and the Flashlight W4D, and at 90 cm there was a strong correlation between the W4DApp and the Flashlight W4D. These results are all expectedly similar between methods because the W4DApp interface was designed display an identical target presentation to the Flashlight W4D. Collectively, these findings indicate that the W4DApp is equally likely to produce interpretable, stable and valid measures of suppression zone size as the Flashlight W4D.

We found a significant negative proportional bias in the 90 cm W4DApp measures of suppression zone size relative to the Flashlight W4D, with an average 0.9° smaller measurement zone with the W4DApp. With large suppression zone size measurements, the W4DApp measures significantly smaller scotoma sizes than the Flashlight W4D. This trend is especially obvious beyond 4° where the Flashlight W4D is testing for peripheral fusion at viewing distances of 50 cm or closer.^7^ The negative bias agrees with the difference in classification observed between devices, with the W4DApp determining a smaller size of suppression zone than the Flashlight W4D technique in 6 BV-abnormal. The W4DApp smaller zone calculation may reflect the more continuous nature of the zone size determination, which permits finer tuning of the size measure. W4Dapp easily uses more discrete steps to vary the visual angle of dot separation than the Flashlight W4D technique. A further confounding factor may arise from the change in the dots visual angle size that occurs with the Flashlight W4D method when the viewing distance is altered, with the visual angle subtended increasing with nearer viewing distance. In contrast, the W4DApp dot size was held at 5 mm diameter for all tests, and this difference between the two assessments may be a source of variation in participant responses. Additionally, the observed negative bias may be due to change in accommodation or vergence at the different viewing distances used with the Flashlight W4D test influencing sensory fusion. As the flashlight is brought closer to the participant, the perceived target size increases, which can affect accommodation.^21^ Although the W4D lights are not considered a highly accommodative target, the awareness of nearness of the flashlight could drive accommodative responses.^21^ Reducing the flashlight viewing distance also affects vergence posture, which can vary significantly in strabismic subjects with high AC/A ratios, such as convergence excess estropia.^21^ By maintaining a fixed visual angle of dot and fixed viewing distance, the W4DApp controls for the potential influence of accommodative/vergence factors on suppression zone size measure that might influence outcomes determined with the Flashlight W4D method.

We found significantly higher success rate and reliability in the determination of depth of suppression by the W4DApp compared with that determined by Flashlight W4D and NDF. Other studies have similarly reported limited test-retest reliability of the Flashlight W4D with NDF method.^12^ Potentially the improved success rate and reliability with W4DApp is due to the continuous nature of the measures, which allow the contrast to be dropped in small numerical increments, whereas the Flashlight W4D is limited by the ordinal measures of the filter bar, with only 6 density intervals available.^10^^,^^11^

Despite favorable success rate and reliability, the W4DApp measures of suppression depth were found to have no absolute agreement or significant correlation with the current clinical Flashlight W4D + NDF or the laboratory-based CBI techniques. In comparison, the established ocular dominance tests Flashlight W4D + NDF and CBI were shown to highly correlate. The significant correlation between the CBI and the flashlight W4D + NDF outcomes suggests that the difference in stimulus configuration between CBI letters and W4D red/green dots is unlikely to be the source of poor agreement between outcomes of the W4DApp and the CBI test.

The method of reducing dot intensity for calculating the interocular contrast ratio (suppression depth) differs between the flashlight and App version W4D tests. The W4DApp measures suppression depth by lowering the alpha opaqueness value of the dot targets seen by the dominant eye and thus lowers central relative contrast without altering peripheral luminance, whereas the Flashlight W4D uses the NDF to lower the luminance of the entire visual field of the dominant eye until simultaneous perception is achieved. Ocular dominance changes that are induced by introduction of an NDF before one eye depend not only on the induced interocular luminance ratio but also on absolute luminance.^22^ Thus the overall illumination in the room and differences in method of varying dot intensity may underlie the poor agreement in depth of suppression determination between the Worth 4 Dot methods.

Although the App design was based on the classical Worth 4 Dot, with care taken to match the spectral characteristics of the App dots to the band-pass of the Good-lite Flashlight W4D red/green glasses, the absolute luminance differs in between the iPad and the Flashlight W4D, and there is difference in the relative luminance between the white dots on the Flashlight W4D and the iPad App. The Flashlight W4D lights were overall brighter. Although the red/green interocular brightness ratio was similar, with green approximately 5 times the brightness of the red in both tests, the white dot of the Flashlight is 20 times brighter than the red, compared with only 5 times brighter with the App. How the white dot that is visible to both eyes acts as a “fusion lock” and whether its relative brightness influences responses is yet to be determined. Previous studies report a difference in fusion response with lighting conditions and between red/green and achromatic polarized versions of the Worth 4 Dot test.^3^^,^^6^^,^^23^ This has been attributed to red/green filters introducing artifacts that degrade or enhance performance in tests of binocularity.^6^ The red/green filters induce binocular retinal rivalry and contribute to dissociation of unstable binocular vision.^24^ In addition, the difference in luminance between the red and the green dots, with the green approximately twice the brightness of the red dots in both tests, could vary the fusion response dependent on whether the amblyopic eye was viewing through the green or the red filter. Following convention, all tests of fusion in this study were conducted with the red filter over right eye and the luminance of the dots was not balanced on the App. A future modification to be tested will be luminance balancing between colors to control for the inter-ocular brightness difference in the dichoptic dot presentation. That is, testing responses where the alpha opaqueness is adjusted to set equal luminance of dots through the red/green glasses.

In this study we did not screen participants for color vision anomalies. Although abnormal color vision does not preclude participants from completing the test,^25^ any participants with protan-type defects will see the red targets as much dimmer than normal, and this will affect suppression depth measurements in the two W4D tests.

Our study does have important limitations, including the limited cohort of participants. Further evaluation is planned in a larger sample size that includes children aged less than seven years. Proposed modifications to the App include incorporation of automatic brightness setting at startup, a staircase bracketing method to determine suppression breakpoints,^19^ and an option to vary the relative luminance of the dots using the brightness adjustment attribute of an iOS device. Currently the dots are superimposed on a black background and so fade to black as they become less opaque. We plan to test a version that incorporates an illuminant white dot behind the colored dots, and use both alpha and brightness adjustments to produce a greater range of dot contrast. We plan to test varying the dot diameter for participants who are limited by low visual acuity, and program the application with shape targets to improve success rate on young children.^8^^,^^26^

Overall, the W4DApp is a viable version of the Worth 4 Dot suppression test that is a convenient and accessible alternative to current flashlight or projection versions for suppression zone size evaluation. Further modifications to the current iteration are planned to improve the determination of suppression depth.

## Appendix 1. Appendix 1. Abnormal Binocular Vision Participant Details

**Table:**

Classification	Age (y)	Right eye visual acuity (logMAR)	Left eye visual acuity (logMAR)	Interocular difference in visual acuity (logMAR)	Randot Stereoacuity(arcsec)	Flashlight W4D at 6 m	Amblyopic treatment
Anisometropic	21	0.00	0.00	0.00	60	RE Suppression	
Anisometropic	37	−0.18	−0.10	0.08	40	Normal	
Anisometropic amblyope	24	0.00	0.40	0.40	100	RE Suppression	
Anisometropic amblyope	13	0.70	0.00	0.70	100	RE suppression	3D computer training
Strabismic	20	−0.02	−0.02	0.00	40	Normal	
Strabismic	46	0.00	0.00	0.00	400	Normal	
Strabismic	21	−0.10	−0.08	0.02	40	Normal	
Strabismic	61	0.00	−0.02	0.02	Nil	RE suppression	
Strabismic	47	0.08	0.12	0.04	Nil	LE suppression	
Strabismic	24	−0.02	0.04	0.06	400	LE suppression	EOM surgery, patching
Strabismic	21	−0.08	0.00	0.08	Nil	LE suppression	EOM surgery
Strabismic	48	0.04	0.14	0.10	Nil	LE suppression	Patching
Strabismic	20	0.02	−0.10	0.12	60	Normal	EOM surgery, patching
Strabismic amblyope	20	0.20	0.00	0.20	Nil	RE suppression	EOM surgery, patching
Strabismic amblyope	20	0.00	0.20	0.20	Nil	LE suppression	
Strabismic amblyope	23	0.00	0.40	0.40	Nil	LE suppression	EOM Surgery, Patching
Strabismic and anisometropic	15	0.00	−0.08	0.08	Nil	RE suppression	Patching, atropine drops
Strabismic and anisometropic amblyope	12	0.40	0.12	0.28	Nil	RE suppression	Patching
Strabismic and anisometropic amblyope	47	0.84	−0.10	0.94	Nil	RE suppression	Patching

Participants are grouped by classification and sorted by increasing interocular difference in visual acuity.
